# Subcutaneous Allergen‐Specific Immunotherapy for Allergic Rhinitis: Divergent IgA Responses in Nasal Mucosa and Blood With Validation of B Cell Class‐Switching in Lymph Nodes and Blood

**DOI:** 10.1002/clt2.70097

**Published:** 2025-09-02

**Authors:** Maryam Jafari, Marianne Petro, Eirini Paziou, Eric Hjalmarsson, Agnetha Karlsson, Monika Ezerskyte, Laila Hellkvist, Susanna Kumlien Georén, Eduardo I. Cardenas, Lars‐Olaf Cardell

**Affiliations:** ^1^ Division of Ear, Nose, and Throat Diseases Department of Clinical Science Intervention and Technology Karolinska Institutet Stockholm Sweden; ^2^ Department of Otorhinolaryngology Karolinska University Hospital Stockholm Sweden

**Keywords:** allergic rhinitis, B cells, IgA, IgG4, lymph node, SCIT

## Abstract

**Background:**

Subcutaneous immunotherapy (SCIT) has been a cornerstone treatment for allergic rhinitis (AR) for over 50 years, consistently demonstrating symptom reduction and modulation of immune responses. Despite this, the underlying mechanisms responsible for the efficacy of SCIT remain incompletely understood, especially with regard to local immune responses in lymph nodes and nasal mucosa.

**Aim:**

To determine the impact of SCIT treatment on immunoglobulin production in blood and nasal mucosa, as well as B cell class‐switching in blood and lymph nodes.

**Methodology:**

Blood, nasal lavage (NAL), and fine‐needle aspirates (FNA) of inguinal lymph nodes were collected from 23 patients with birch and/or timothy pollen‐induced AR before and after SCIT updosing. General response to SCIT was evaluated with symptom and medication scores, as well as allergen‐mediated activation of basophils. Allergen‐specific IgE, IgA, IgG, and IgG4 were measured in blood and NAL via ELISA. B‐cell class‐switching was assessed in blood and FNAs by flow cytometry.

**Results:**

AR symptoms, medication use, and basophil sensitivity to allergens was reduced after SCIT updosing. Interestingly, the plasma levels of allergen‐specific IgA, IgG, and IgG4 increased after SCIT updosing, while the levels of allergen‐specific IgE and IgA decreased in NAL at this timepoint. Moreover, these results were accompanied by an increase in conventional (IgD^−^CD27^+^) and unconventional (IgD^−^CD27^−^) memory B cells in blood and lymph nodes, respectively.

**Conclusion:**

This study highlights the differential effects of SCIT on local and systemic immunity and identifies early immunological changes associated with treatment. However, confirmation of long‐term tolerance will require extended follow‐up, including in‐season analyses. The local decrease in allergen‐specific IgA in NAL, alongside a systemic increase in allergen‐specific IgA and IgG4, underscores the importance of assessing both mucosal and systemic responses when evaluating SCIT efficacy.

## 1 Introduction

Subcutaneous immunotherapy (SCIT) has been a cornerstone treatment for allergic rhinitis (AR) for more than 5 decades, effectively reducing symptoms and altering immune responses. SCIT notably induces a shift in the production of allergen‐specific immunoglobulins that favors other classes over IgE [1, 2, 3]. Specifically, SCIT has long been known to cause an increase in allergen‐specific IgG4 in blood [4], and two recent studies report that it can also result in enhanced production of allergen‐specific IgG4 in the nose [5, 6]. These outcomes are thought to be crucial for SCIT efficacy given that allergen‐specific IgG4 can inhibit IgE‐mediated immune responses via allergen interception, as well as FcγRIIb crosslinking on the surface of mast cells and basophils [7, 8].

Interestingly, recent studies suggest that SCIT also may tigger a small production of allergen‐specific IgA in both blood and nasal mucosa [5, 9]. Nevertheless, allergen‐specific IgA could play contradictory roles at these anatomical sites and its role in SCIT is not well characterized. In fact, despite extensive research on the systemic effects of SCIT, our understanding of its impact on immunoglobulin production in the nose remains limited. Moreover, the impact of SCIT on B cell class‐switching within human lymph nodes is critical yet underexplored.

The present study aimed to address these gaps by assessing immunoglobulin production in blood and nasal lavage (NAL), as well as B cell class‐switching in blood and lymph nodes, in samples from patients before and shortly after SCIT updosing.

This study was designed as an exploratory, mechanistic investigation to characterize early immunologic changes following SCIT updosing, with a focus on compartment‐specific responses rather than testing a single directional hypothesis.

Our results shed light on how local and systemic immunity may collaborate in the early immunological changes associated with allergen immunotherapy.

## 2 Materials and Methods

### 2.1 Patient Characteristics

Twenty‐three patients with moderate to severe birch and/or timothy pollen‐induced seasonal AR were included in the present study (Table 1). Inclusion criteria consisted of a confirmed clinical history of seasonal allergy to birch and/or timothy pollen, a positive skin prick test with a wheal diameter of ≥ 3 mm for the relevant allergen(s), and a serum concentration of allergen‐specific IgE ≥ 0.35 kU/mL. In addition, all patients had a Rhinoconjunctivitis Total Symptom Score (RTSS) greater than 8 despite the use of antihistamines during the previous pollen season.

**TABLE 1:** Patient characteristics.

	Patients (*N* = 23)
Age^a^	34 (18–50)
Gender, *n* (%)	
Male	11 (48%)
Female	12 (52%)
Allergy	
Only birch, *n* (%)	4 (17%)
Only timothy, *n* (%)	5 (22%)
Both birch and timothy, *n* (%)	14 (61%)
Asthma, *n* (%)	4 (17%)

^a^ Median (range), N/A, not applicable.

Mild seasonal or exercise‐induced asthma was permitted if symptoms were well controlled with short‐acting β2‐agonists, FEV1 exceeded 70% of predicted, and bronchodilator reversibility was < 12%, as confirmed by baseline spirometry. Patients underwent thorough clinical evaluation and spirometry testing to rule out uncontrolled or comorbid asthma.

Exclusion criteria included perennial or uncontrolled asthma, other pulmonary diseases, autoimmune or collagen vascular disease, chronic infections, severe atopic dermatitis, food allergy, use of beta blockers or ACE inhibitors, symptomatic sensitization to house dust mite or furry animals with daily exposure, chronic upper airway disease, recent upper respiratory tract infection (≤ 2 weeks), recent corticosteroid use (≤ 2 months), antihistamine intake within 24 h prior to sampling, history of chronic rhinosinusitis with or without nasal polyposis, pregnancy or breastfeeding, planned pregnancy, inability to register symptoms electronically, obesity (BMI > 30), or withdrawn informed consent. Patients with known immune‐related comorbidities were also excluded to reduce immunologic confounding.

All participants received SCIT using ALK‐registered allergen extracts (Alutard SQ, ALK‐Abelló, Denmark) for birch and/or timothy grass pollen, based on their individual sensitization profiles. In this study, assessment of localized immunologic alterations with changes in the cell subsets of the draining lymph nodes after subcutaneous injections was in focus. Since fine needle aspirations are easily performed on superficial inguinal lymph nodes, the SCIT treatment was administered in the thighs. The injections and samplings were consistently performed on the same side throughout treatment: birch pollen in the right thigh and grass pollen in the left thigh, with corresponding lymph node analysis. For patients sensitized to both allergens (*n* = 14), both treatments were given simultaneously in their respective thighs.

The SCIT protocol followed national guidelines, with a 7‐week updosing schedule for monosensitized patients and an 11‐week schedule for dual‐sensitized patients, followed by maintenance injections every 6–8 weeks for a total duration of 3–4 years.

### 2.2 Study Design

**FIGURE 1:**
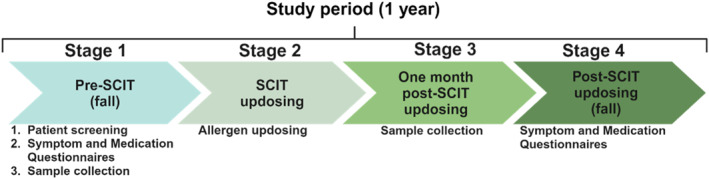
Study design. During stage 1, patients were screened for inclusion, samples (blood, NAL, lymph node FNAs) were collected, and patients completed questionnaires regarding symptoms and medication usage during the previous pollen season. In stage 2, patients received SCIT updosing. Approximately 1 month after post‐SCIT updosing (stage 3) and before the start of the pollen season, samples (blood, NAL, lymph node FNAs) were collected again. During stage 4, which took place in the fall after the pollen season, patients filled out questionnaires regarding symptom severity and medication usage during their first pollen season post‐SCIT updosing.

The study was conducted at the Department of Otorhinolaryngology, Karolinska University Hospital, Stockholm, Sweden, and consisted of four stages, summarized in Figure 1. The inclusion visit (stage 1) took place in the fall after the birch and timothy pollen seasons. During this stage, patients were screened for birch and/or timothy‐induced seasonal AR and asked to fill out questionnaires regarding symptom severity and medication usage during the previous allergen season. In addition, patients provided blood, NAL (*more detail about NAL collection see* Supporting Information S1), and fine‐needle aspirates (FNAs) of inguinal lymph nodes. All information and samples obtained during stage 1 will henceforth be referred to as pre‐SCIT.

During the second stage, a 7‐ or 11‐week updosing regimen of SCIT for birch and/or timothy pollen was administered to patients according to standard protocols. In brief, SCIT was administered in patients' thighs and a step‐wise increase in allergen dose for each visit was used until all patients reached a final maintenance dose of 100,000 SQ‐U birch and/or timothy pollen.

The third stage took place approximately 1 month (4–6 weeks) after completion of stage 2, before the following start of maintenance dosing, and before the start of the pollen seasons. During this stage, patients provided blood, NAL, and lymph node FNAs again. All information and samples obtained after completion of stage 2 will subsequently be referred to as post‐SCIT updosing. Finally (stage 4), patients came back to the clinic after the pollen season (exactly 1 year after their inclusion visit) and were asked to fill out questionnaires regarding symptom severity and medication usage during their first pollen season post‐SCIT updosing.

### 2.3 Symptom Severity and Medication Usage Scores

Symptom severity was monitored using the RTSS and visual analog scale (VAS) questionnaires. The RTSS rates four nasal symptoms (sneezing, rhinorrhea, congestion, and itching) and two ocular symptoms (tearing and itching) on a scale of 0–3, with higher values indicating more severe symptoms and a maximum possible score of 18 [10]. The VAS rates the same four nasal symptoms as the RTSS on a scale of 0–10, with higher values indicating more severe symptoms and a maximum possible average score of 10 [11]. A modified medication usage score [12] was used to quantify the severity of medication usage as follows: oral antihistamines (1 point), local nasal treatment excluding cortisone (1 point), local eye treatment (1 point), inhaled bronchodilator (1 point), topical nasal cortisone (2 points), inhaled cortisone (2 points), leukotriene receptor antagonist (2 points), and omalizumab or cortisone administered as tablets, eye drops, or injections (4 points) for a maximum possible score of 14. The RTSS, VAS, and the modified medication score are well‐established and widely applied tools in SCIT research. These instruments are supported by regulatory guidance (CHMP/EWP/18504/2006; 2008; 6) and are recommended by international expert groups such as ARIA (Allergic Rhinitis and its Impact on Asthma) for evaluating clinical outcomes in allergic rhinitis. RTSS and VAS, in particular, are valued for their ease of use, sensitivity, and reliability in capturing patient‐reported symptoms and treatment effects.

### 2.4 Quality of Life Score

In addition to measurements of symptoms and medication usage a simplistic assessment of quality of life (QoL) was performed by asking patients to rate how much their AR symptoms contributed to tiredness, concentration difficulties, and overall life limitations during the pollen season on a scale from 0 to 10. For visualization, the QoL scores were transformed by subtracting the mean of these three components from the maximum value [10], so that higher values reflect better QoL.

### 2.5 Basophil Activation Test

Allergen sensitivity was assessed in pre‐ and post‐SCIT samples by quantifying the response of blood basophils to allergens. For this purpose, whole blood was stimulated with three different allergen concentrations (31.25, 125, and 500 SQU/ml; 30 min, 37°C) and stained with the antibodies listed in Supporting Information S1: Table 1. Red blood cells were lysed (150 mM NH_4_Cl, 10 mM NaHCO_3_, 1 mM EDTA in water; 5 min) prior to flow cytometric analysis. Basophils were identified as IgE^+^HLA‐DR^−^ cells and the percentage of activated (CD63^+^) basophils was quantified for the three different allergen concentrations. These results were plotted and the area under the curve (AUC) was calculated for each patient.

### 2.6 B Cell Analysis

**FIGURE 2:**
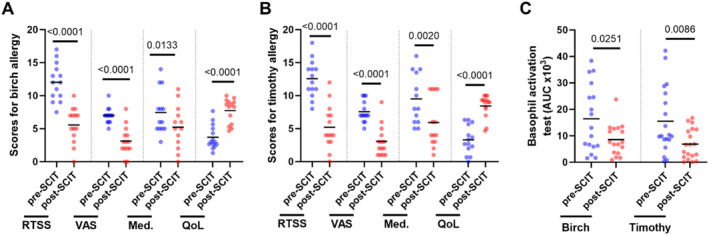
Evaluation of symptom and medication scores, as well as basophil activation test. (A, B) Comparison of rhinoconjunctivitis total symptom score (RTSS), visual analog scale (VAS), medication usage score (Med.), and quality of life score (QoL) regarding the last birch and timothy pollen seasons before SCIT (pre‐SCIT) and the first pollen seasons after SCIT updosing (post‐SCIT) (*n* = 13–14). (C) Comparisons of basophil activation in response to birch (*n* = 17) or timothy (*n* = 19) pollen allergen in samples taken before SCIT (pre‐SCIT) and 4‐6 weeks after SCIT updosing (post‐SCIT). The values shown represent the area under the curve (AUC) of the percentage of CD63^+^ basophils in response to stimulation with 31.25, 125, and 500 SQU/ml allergen. The lower horizontal lines represent the mean. Statistical comparisons were performed using paired Student's *t*‐test. *p*‐values provided above the upper horizontal lines.

Peripheral blood mononuclear cells (PBMCs) were isolated from whole blood via Ficoll separation. PBMCs and cells from FNAs were Fc‐blocked, stained with the antibodies listed in Supporting Information S1: Table 2, and fixed prior to flow cytometric analysis. B cells were identified as CD19^+^CD20^+^ cells and changes in four subpopulations were quantified: naïve (IgD^+^CD27^−^) conventional memory (IgD^−^CD27^+^), unconventional memory (IgD^−^CD27^−^), and non‐switched memory (IgD^+^CD27^+^). In a subsequent assessment, PBMCs were stained with the antibodies listed in Supporting Information S1: Table 3 to determine changes in B cell (CD19^+^CD20^+^) and plasma cell (CD19^+^CD20^−^) expression of different IgG and IgA subclasses. The gating strategies used are described in Supporting Information S1: Figures 1 and 2.

### 2.7 Flow Cytometry

Flow cytometry was conducted using an LSR Fortessa X20, with data analysis done in FlowJo version 10.7.1 (both from BD Biosciences, Franklin Lakes, NJ, USA).

### 2.8 Immunoglobulin Quantification

Allergen‐specific IgE, IgG, and IgG4 were quantified in blood and NAL samples at the Karolinska University Laboratory. Allergen‐specific IgA was quantified in blood and NAL samples using an in‐house ELISA (could not distinguish between secretory and monomeric IgA) (see Supporting Information S1 Methods for details). Total protein concentration in each sample was determined using a Pierce Bradford Protein Assay Kit (ThermoFisher Scientific, Waltham, MA, USA) and used to normalize the immunoglobulin data.

### 2.9 Ethics Statement

All patients provided written informed consent before inclusion and all procedures and handling of patient information were conducted in accordance with the Declaration of Helsinki and the ethical permit approved by the Swedish Ethical Review Authority (Diary No. 2016/823‐31/2).

### 2.10 Statistical Analysis

Data analysis was performed using GraphPad Prism software (Version 10, San Diego, CA, USA). Pairwise comparisons were performed using Student's t‐test or Wilcoxon signed‐rank test for data that was normally and not normally distributed, respectively. Exploratory Spearman's rank correlation analyses were conducted to assess potential associations between immunologic parameters and clinical outcomes following SCIT. Statistical significance was defined as *p* < 0.05.

## 3 Results

### 3.1 Patient Characteristics

Of the 23 patients, 14 were allergic to both birch and timothy, 4 were allergic to birch but not to timothy, and 5 were allergic to timothy but not to birch. A summary of all patient characteristics can be found in Table 1.

### 3.2 Symptom and Medication Scores, and Basophil Activation Test Analysis

Patients reported a reduction in RTSS, VAS, and medication usage scores, as well as an increase in overall QoL, during the first birch and timothy pollen seasons following SCIT updosing, compared to the last pollen season before treatment (Figure 2A,B). Symptom score data were available for 13–14 of the 19 birch‐ and 19 grass‐allergic individuals, as not all participants provided complete post‐SCIT questionnaire responses. Moreover, the basophil activation test indicated a reduction in the sensitivity to birch and timothy pollen approximately 1 month after SCIT updosing (Figure 2C).

### 3.3 Assessment of Allergen‐Specific Immunoglobulins in Plasma and NAL

**FIGURE 3:**
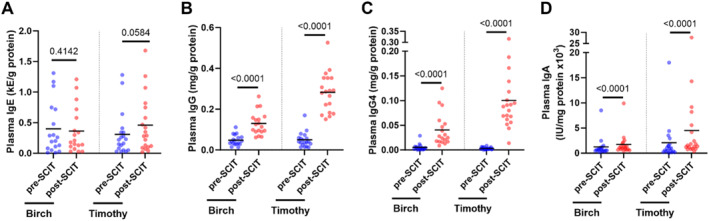
Quantification of allergen‐specific immunoglobulins in plasma. Comparisons of the levels of allergen‐specific (A) IgE, (B) IgG, (C) IgG4, and (D) IgA in plasma samples taken from patients allergic to birch (*n* = 18) and/or timothy (*n* = 19) pollen before SCIT treatment (pre‐SCIT) and 4–6 weeks after SCIT updosing (post‐SCIT). The lower horizontal lines represent the mean. Statistical comparisons were performed using (A) paired Student's *t*‐test and (B–D) Wilcoxon signed‐rank test. *p*‐values provided above the upper horizontal lines.

**FIGURE 4:**
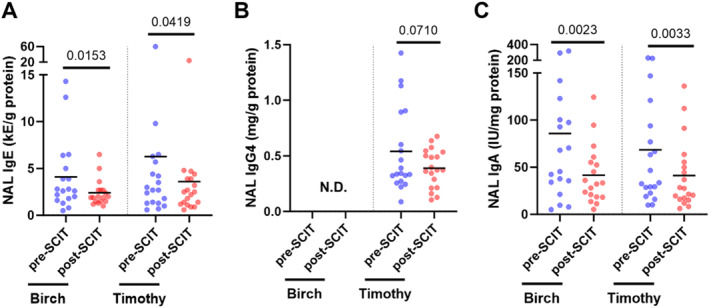
Quantification of allergen‐specific immunoglobulins in NAL. Comparisons of the levels of allergen‐specific (A) IgE, (B) IgG4, (C) IgA in NAL samples taken from patients allergic to birch (*n* = 18) and/or timothy (*n* = 19) pollen before SCIT treatment (pre‐SCIT) and 4–6 weeks after SCIT updosing (post‐SCIT). The lower horizontal lines represent the mean. N.D. = non‐detectable. Statistical comparisons were performed using Wilcoxon signed‐rank test. *p*‐values provided above the upper horizontal lines.

Although there were no statistically significant alterations in the levels of allergen‐specific IgE in plasma approximately 1 month after SCIT updosing (Figure 3A), the plasma levels of allergen‐specific IgG, IgG4, and IgA were all increased at this timepoint (Figure 3B–D). On the other hand, the levels of allergen‐specific IgE and IgA in NAL were reduced after SCIT updosing (Figure 4A,C). Of note, the levels of birch pollen‐specific IgG4 could not be detected in NAL, while the levels of timothy pollen‐specific IgG4 remained unaltered at this location (Figure 4B).

### 3.4 Exploratory Correlation Between Immunologic Markers and Clinical Outcomes

Correlation analyses between RTSS scores and key immunologic parameters, including plasma IgG4 and NAL IgA levels, did not reveal statistically significant associations for either birch or grass allergens.

### 3.5 Analysis of Class‐Switched B Cells in Blood and Lymph Nodes

**FIGURE 5:**
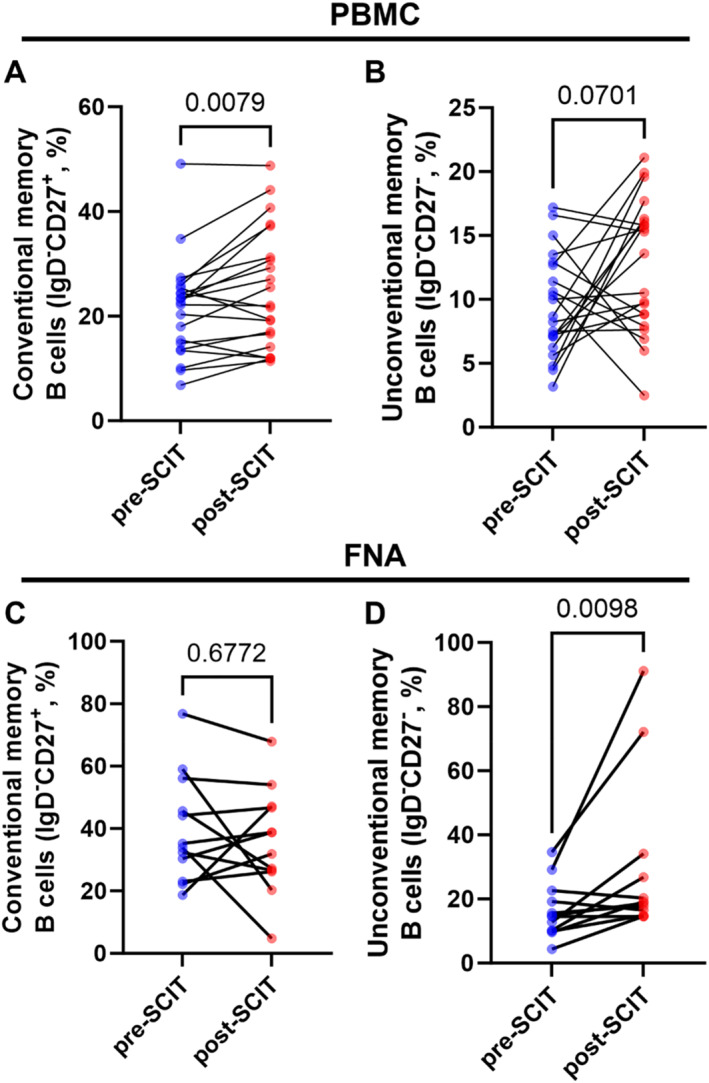
Analysis of conventional and unconventional B cells in PBMC and FNA. Comparisons of the percentage of (A, C) conventional memory B cells (IgD^−^CD27^+^) and (B, D) unconventional memory B cells (IgD^−^CD27^−^) in (A–B) PBMC (*n* = 21) and (C–D) FNA (*n* = 12) samples taken before SCIT (pre‐SCIT) and 4–6 weeks after SCIT updosing (post‐SCIT). Statistical comparisons were performed using Wilcoxon signed‐rank test *p*‐values provided above the horizontal lines.

To determine the impact of SCIT updosing on B cells, the percentages of class‐switched conventional and unconventional memory B cells was determined in blood and FNA samples from SCIT patients. Notably, conventional memory B cells (IgD^−^CD27^+^) increased in blood, but not in FNA samples (Figure 5A,C). On the other hand, unconventional memory B cells (IgD^−^CD27^‐^) increased in FNA samples, but not in blood (Figure 5B,D).

### 3.6 Analysis of IgG and IgA Subclass Expression on the Surface of B and Plasma Cells

**FIGURE 6:**
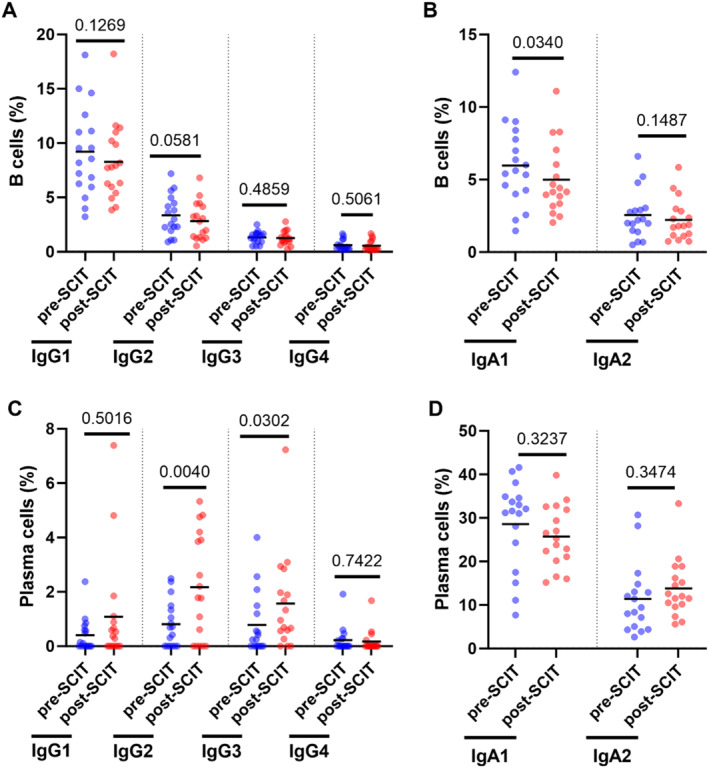
Analysis of IgG and IgA subclass expression on the surface of circulating B and plasma cells. Comparisons of the percentage of (A–B) B cells (CD19^+^CD20^+^) and (C–D) plasma cells (CD19^+^CD20^−^) expressing different (A, C) IgG and (B, D) IgA subclasses in PBMC samples taken before SCIT (pre‐SCIT) and 4–6 weeks after SCIT updosing (post‐SCIT; *n* = 17). Statistical comparisons were performed using (A–B, D) paired Student's *t*‐test and (C) Wilcoxon signed‐rank test. *p*‐values provided above the upper horizontal lines.

Finally, the surface expression of different IgG and IgA subclasses was assessed on both B cells (CD19^+^CD20^+^) and plasma cells (CD19^+^CD20^−^) from PBMC samples of SCIT patients. Circulating B cells expressed unaltered levels of all IgG subclasses and slightly less IgA1 approximately 1 month after SCIT updosing (Figure 6A,B), while circulating plasma cells expressed more IgG2 and IgG3 at this timepoint (Figure 6C). No statistically significant alterations in IgA expression were identified in circulating plasma cells post‐SCIT (Figure 6D).

## 4 Discussion

The effectiveness of SCIT in reducing pollen‐induced AR symptoms is well established. In line with this, the present study indicates that SCIT patients experience a marked reduction in AR symptoms since the first pollen season following SCIT updosing. Moreover, this study indicates that basophil sensitivity to pollen allergens is already reduced shortly after (4–6 weeks) SCIT updosing and before the start of the pollen season. Taken together, these results confirm that SCIT treatment can quickly grant protection against pollen‐induced AR.

Our study aimed to isolate early treatment‐associated effects prior to environmental allergen exposure. Therefore, the observed immunological changes should be interpreted as SCIT‐associated alterations in the B cell compartment, rather than as definitive markers of long‐term immunological tolerance. This gap is important for understanding the timeline of immune adaptation during SCIT. Since sampling was conducted outside the pollen season, natural allergen avoidance may have acted as a confounding factor. Furthermore, the absence of a non‐treated allergic rhinitis control group remains a key limitation. However, prior studies in untreated AR patients have shown persistent B cell dysregulation off‐season, which was not observed in our SCIT‐treated cohort, supporting the treatment‐associated nature of the observed changes [13]. Future longitudinal studies including untreated controls will be essential to determine whether these changes persist over time and to explore their possible role in promoting immune tolerance.

To further explore the relationship between immune modulation and clinical improvement, we performed exploratory Spearman correlation analyses between RTSS scores and key immunologic markers. While no statistically significant associations were found, the results suggest that clinical and immunologic responses may develop along distinct timelines in the early phase of SCIT.

Although the mechanisms behind the proven efficacy of SCIT are not completely understood, allergen‐specific IgG antibodies, particularly of the IgG4 subclass, have been forwarded as likely key factors to success key factors to success. Production of allergen‐specific IgG4 has long been known to increase systemically after SCIT treatment [4], and more recent studies indicate that it may also increase in the nasal mucosa during natural pollen season [6] or after years of SCIT treatment [5]. Notably, binding of IgG4 to allergens can impede their recognition by IgE, thus preventing allergic responses [14]. Moreover, studies on murine models indicate that allergen‐bound IgG4 can inhibit the activation of mast cells and basophils via FcγRIIb crosslinking [7, 8]. In line with the established literature, we now report a pronounced increase in the systemic levels of allergen‐specific IgG and IgG4 shortly after (4–6 weeks) SCIT updosing and before the start of the pollen season. Nevertheless, we found that the levels of allergen‐specific IgG4 in NAL were undetectable (birch) or unaltered (timothy) at this timepoint. We normalized antibody levels to total protein levels to adjust for differences in sample volume, especially in nasal lavages. NAL has much lower protein content than plasma, including lower levels of albumin and serum proteins [15, 16]. This may affect how well the results can be compared to NAL and plasma samples.

Birch‐specific IgG4 was likely not detected in NAL due to the early stage of SCIT treatment. Previous studies have shown that increases in allergen‐specific IgG4 in the nasal mucosa typically occur after prolonged treatment or during natural allergen exposure [17]. The reduced basophil sensitivity to allergens observed shortly after SCIT updosing, could underscore the importance of systemic allergen‐specific IgG4 in alleviating AR symptoms. This is consistent with earlier findings demonstrating that IgG4 can inhibit basophil and mast cell activation through engagement of the inhibitory FcγRIIb receptor [18].

Two recent studies suggest that SCIT can also trigger a small increase in the production of allergen‐specific IgA in blood [5, 9], and one of them also identified a small and transient increase in allergen‐specific IgA in the nasal mucosa following 1‐year of SCIT treatment [5]. In the present study, we report an increase in the plasma levels of allergen‐specific IgA together with a decrease in the levels of these antibodies in NAL shortly after (4–6 weeks) SCIT updosing. These results suggest that secretory IgA (sIgA) and monomeric IgA, the most prevalent forms of IgA in the nasal mucosa and blood, respectively, might play opposing roles at this early timepoint. Indeed, whereas sIgA is a potent inducer of eosinophil and basophil degranulation [19, 20, 21, 22], monomeric IgA can inhibit the activation of basophils and mast cells [22]. Moreover, allergen‐specific IgA is known to increase in the nasal mucosa during natural pollen season in AR patients [23], increased levels of these antibodies have been associated with heightened AR symptoms [24], and we now report that AR patients have significantly higher levels of allergen‐specific IgA in NAL before SCIT treatment. Considering the potential pro‐inflammatory effects of sIgA in the nasal mucosa, a reduction in allergen‐specific IgA in NAL might indicate a decrease in local inflammation. Although our IgA ELISA could not distinguish between secretory and monomeric IgA, our results are compatible with a protective reduction in pro‐inflammatory sIgA in the nose and an increase in anti‐inflammatory monomeric IgA in the blood at this early timepoint. Our data do not directly demonstrate isoform‐specific effects. Rather, the observed changes are consistent with differential roles of IgA subclasses described in the literature, but further studies using methods capable of distinguishing between sIgA and monomeric IgA are needed to confirm this. Still, it is important to recognize that these findings may not fully reflect mucosal responses under natural allergen exposure conditions.

This study is limited by a relatively small sample size, particularly for invasive procedures such as lymph node FNAs (*n* = 12), which may restrict statistical power and generalizability. However, it is important to underscore that fine needle aspiration of lymph nodes in the context of allergen immunotherapy is exceptionally rare in human studies, especially during early treatment phases. These unique samples provide valuable insight into early systemic immune responses at a peripheral site of antigen exposure following SCIT. While the inguinal lymph nodes do not directly reflect nasal mucosal immunity, they may serve as a model for early systemic B cell priming rather than local mucosal events. Accordingly, claims regarding mechanistic insight have been interpreted with caution. As an exploratory, mechanistic study, our primary goal was to generate hypotheses and assess feasibility, laying the groundwork for future investigations in larger, powered cohorts.

A key benefit of SCIT is that it can offer long‐lasting allergen tolerance, for which induction of immunological memory is required [25]. In the present study, we report an increase in circulating conventional memory B cells (IgD^−^CD27^+^) shortly after (4–6 weeks) completion of SCIT updosing. Moreover, we show an increase in unconventional memory B cells (IgD^−^CD27^−^) in inguinal lymph nodes at this timepoint. To understand these seemingly divergent results, it is important to consider that although both memory B cell subsets are class‐switched and antigen‐experienced, unconventional memory B cells can represent an exhausted B cell phenotype that arises after chronic antigen stimulation [26]. However, recent studies suggest that IgD^−^CD27^−^ B cells represent a heterogeneous population with diverse functional potential. In cancer and inflammatory conditions, for example, they have been associated with regulatory or effector functions, and in some contexts display memory‐like properties [27, 28].

This could explain why we see an increase in unconventional memory B cells in the lymph nodes adjacent to the site of allergen administration following SCIT updosing. Taking all things together, these results indicate an on‐going engagement in local lymph nodes that ultimately could lead to the development of circulating conventional memory B cells.

Finally, our analysis on circulating B and plasma cell expression of different IgG and IgA subclasses revealed a slight reduction in IgA1 B cells, as well as a pronounced increase in IgG2 and IgG3 plasma cells shortly after (4–6 weeks) SCIT updosing. Although little is known regarding the roles of IgG2 and IgG3 in allergy, a study suggests that they might have a similar role in allergen tolerance as IgG4 [29]. Of note, these results represent alterations in global B and plasma cell populations, and it is possible that an analysis focused on allergen‐specific cells, which represent a small fraction, might have yielded different results. This could explain why we observed a marked increase in allergen‐specific IgG4 and IgA antibodies in plasma, yet did not detect a corresponding increase in IgG4^+^ or IgA^+^ B cell subsets. We acknowledge this as a limitation of the study. Future studies should employ more sensitive approaches such as allergen‐tetramer‐based flow cytometry or single‐cell sequencing to track antigen‐specific B cell clones and better understand these differences. Moreover, it is unclear whether these alterations in IgG and IgA subclass expression on circulating B and plasma cells represent a temporary result or a long‐lasting effect, given that we did not include memory markers in this final analysis.

## 5 Conclusion

In summary, this study provides insights into early immunological responses to SCIT, particularly regarding basophil sensitivity, immunoglobulin modulation, and B cell class‐switching across distinct anatomical compartments. The observed increase in allergen‐specific IgG4 and IgA in blood, alongside a reduction in allergen‐specific IgE and IgA in the nasal mucosa, indicates early SCIT‐associated immunomodulation, even in the absence of nasal IgG4. Furthermore, we report evidence of early memory B cell formation, which may be relevant for long‐term immunological outcomes. These findings underscore the importance of assessing both local and systemic immune responses when evaluating SCIT efficacy. Importantly, while the inclusion of lymph node FNA samples provides rare access to early immune events following subcutaneous allergen exposure, these data should be interpreted as reflecting early systemic B cell priming rather than mucosal immunity. As such, they contribute to a broader understanding of SCIT‐induced immune modulation in humans.

## Author Contributions

**Maryam Jafari:** conceptualization, methodology, formal analysis, data curation, resources, project administration, visualization, writing – review and editing, writing – original draft, investigation, validation, software. **Marianne Petro:** resources, writing – review and editing, methodology. **Eirini Paziou:** resources, writing – review and editing. **Eric Hjalmarsson:** conceptualization, data curation, investigation, methodology, validation, resources, writing – review and editing, software, formal analysis. **Agnetha Karlsson:** resources, writing – review and editing. **Monika Ezerskyte:** writing – review and editing. **Laila Hellkvist:** resources, writing – review and editing. **Susanna Kumlien Georen:** conceptualization, investigation, methodology, project administration, writing – review and editing. **Eduardo I. Cardenas:** data curation, formal analysis, validation, writing – review and editing. **Lars‐Olaf Cardell:** conceptualization, methodology, project administration, supervision, writing – review and editing, funding acquisition.

## Ethics Statement

The local Ethical Committee approved the study in Stockholm, Sweden (2008‐1278‐31, 2015‐1748‐32). All participants gave their written, informed consent. All procedures were conducted according to the principles expressed in the Declaration of Helsinki.

## Conflicts of Interest

The authors declare no conflicts of interest.

## Supporting information


Supporting Information S1

## Data Availability

The datasets during and/or analyzed during the current study are available from the corresponding author upon reasonable request.
