# Supplementary material for: Subcutaneous Allergen‐Specific Immunotherapy for Allergic Rhinitis: Divergent IgA Responses in Nasal Mucosa and Blood With Validation of B Cell Class‐Switching in Lymph Nodes and Blood
Source: Clin Transl Allergy. 2025 Sep 2;15(9):e70097. doi: 10.1002/clt2.70097 (PMC12403106; doi:10.1002/clt2.70097)
Supplement: Supplementary file 1 — Supporting Information S1 [file CLT2-15-e70097-s001.docx]

**Supplementary Methods**

*IgA quantification*

To quantify IgA in plasma and NAL, 96-well plates were coated overnight at room temperature with Aquagen SQ Birch or Aquagen SQ Timothy (both from ALK-Abello, Hörsholm, Denmark) in coating buffer (44 mM NaHCO3 and 6 mM Na2CO3 in water; pH 9.5). Unbound allergen was removed by rinsing with wash buffer (0.05% Tween-20 in PBS) and plates were incubated in blocking solution (1h, room temperature, 1% bovine serum albumin fraction V in wash buffer). Following rinsing, plates were incubated (2h, room temperature) with samples (plasma: 1:10 dilution in blocking buffer, NAL: undiluted or 1:2-4 in blocking buffer). Sample-containing wells were rinsed with wash buffer and plates were incubated (1h, room temperature) with biotinylated goat-anti-human IgA antibody (1:20000 in blocking buffer; Thermo Fisher Scientific, Waltham, MA, USA). Unbound antibody was rinsed away with wash buffer, and plates were incubated (20 min, room temperature) with streptavidin-HRP (1:200 in blocking buffer; R&D Systems, Minneapolis, MN, USA). Unbound streptavidin was rinsed away with wash buffer, and plates were incubated (10 min, room temperature) with tetramethylbenzidine substrate solution (R&D Systems). The substrate reaction was stopped with 2N sulfuric acid and the plate was immediately read (450 nm) using a Multiskan EX plate reader (Thermo Fisher Scientific). To generate a standard curve, a sample with high concentration of allergen-specific IgA (high absorbance) was chosen and 1:2 serial dilutions were prepared and run as described above (1:20, 1:40, 1:80, 1:160, 1:320, 1:640, 1:1280, 1:2560).

*Nasal Lavage (NAL) Collection*

NAL samples were collected using isotonic saline solution (NaCl) dispensed via a nasal spray pump. Patients were first instructed to gently blow their nose. After that 3–4 sprays of saline were administered into one nostril while avoiding direct spray onto the nasal septum to minimize the risk of bleeding. With the patient seated and slightly leaning forward over a sterile funnel placed in a chilled 15 mL collection tube, effluent was allowed to drip passively into the funnel. If bleeding occurred, the procedure was paused, and, if safe, resumed in the opposite nostril using a new tube. After collecting approximately 3.5 mL per nostril (total 7 mL), the samples were kept on ice and processed within 30 minutes *Centrifugation and Sample Handling*

NAL samples were centrifuged at 300 × g for 10 minutes at 5°C. The supernatant (containing soluble immunoglobulins) was carefully transferred to a clean tube, supplemented with 10 µL protease inhibitor, gently mixed, and aliquoted into five Eppendorf tubes for storage at -80°C. The cell pellet was resuspended in 500 µL of RNAprotect Cell Reagent, transferred to a separate tube, incubated at 4°C for 16-24 hours, and then frozen at -80°C for downstream RNA analyses.

***Note on Methodological Considerations:***

Although the described NAL protocol is widely used in clinical research, it has not been formally validated for the detection of allergen-specific immunoglobulins in the absence of natural allergen exposure. The timing of sample collection outside the pollen season may also have influenced the ability to detect subtle mucosal immune responses. These factors should be considered when interpreting the nasal antibody data.

**Supplementary Tables**

**Supplementary Table 1.** Antibodies used in basophil activation test.

| **Target** | **Fluorophore** | **Clone** | **Company** |
| --- | --- | --- | --- |
| **CD63** | PerCP-Cy 5.5 | H5C6 | BD Biosciences |
| **IgE** | PE | MHE-18 | Biolegend |
| **HLA-DR** | PE-Cy7 | G46-6 | BD Biosciences |

**Supplementary Table 2.** Antibodies and live/dead staining used in B cell class-switching analysis.

| **Target** | **Fluorophore** | **Clone** | **Company** |
| --- | --- | --- | --- |
| **CD19** | BB700 | SJ25C1 | BD Biosciences |
| **CD20** | Alexa Fluor® 700 | 2H7 |  |
| **IgD** | APC-H7 | IA6-2 |  |
| **CD27** | PE-Cy™7 | M-T271 |  |
| **Live/Dead** | APC | NA | Invitrogen |

**Supplementary Table 3.** Antibodies and live/dead staining used in B and plasma cell IgG and IgA subset analysis.

| **Target** | **Fluorophore** | **Clone** | **Company** |
| --- | --- | --- | --- |
| **CD19** | BUV395 | 1D3 | BD Biosciences |
| **CD20** | Alexa Fluor® 700 | 2H7 |  |
| **IgM** | BV510 | G20-127 |  |
| **IgD** | BV421 | IA6-2 |  |
| **IgG1/IgG2** | PE | SAG1/SAG2 | Cytogonos |
| **IgG2/IgG3** | FITC | SAG2/SAG3 |  |
| **IgA1/IgA2** | PerCP-Cy 5.5 | SAA1/SAA2 |  |
| **IgA1/IgG4** | APC | SAA1/SAG4 |  |
| **Live/Dead** | APC-Cy7 | NA | Invitrogen |

**Supplementary Figures**

**
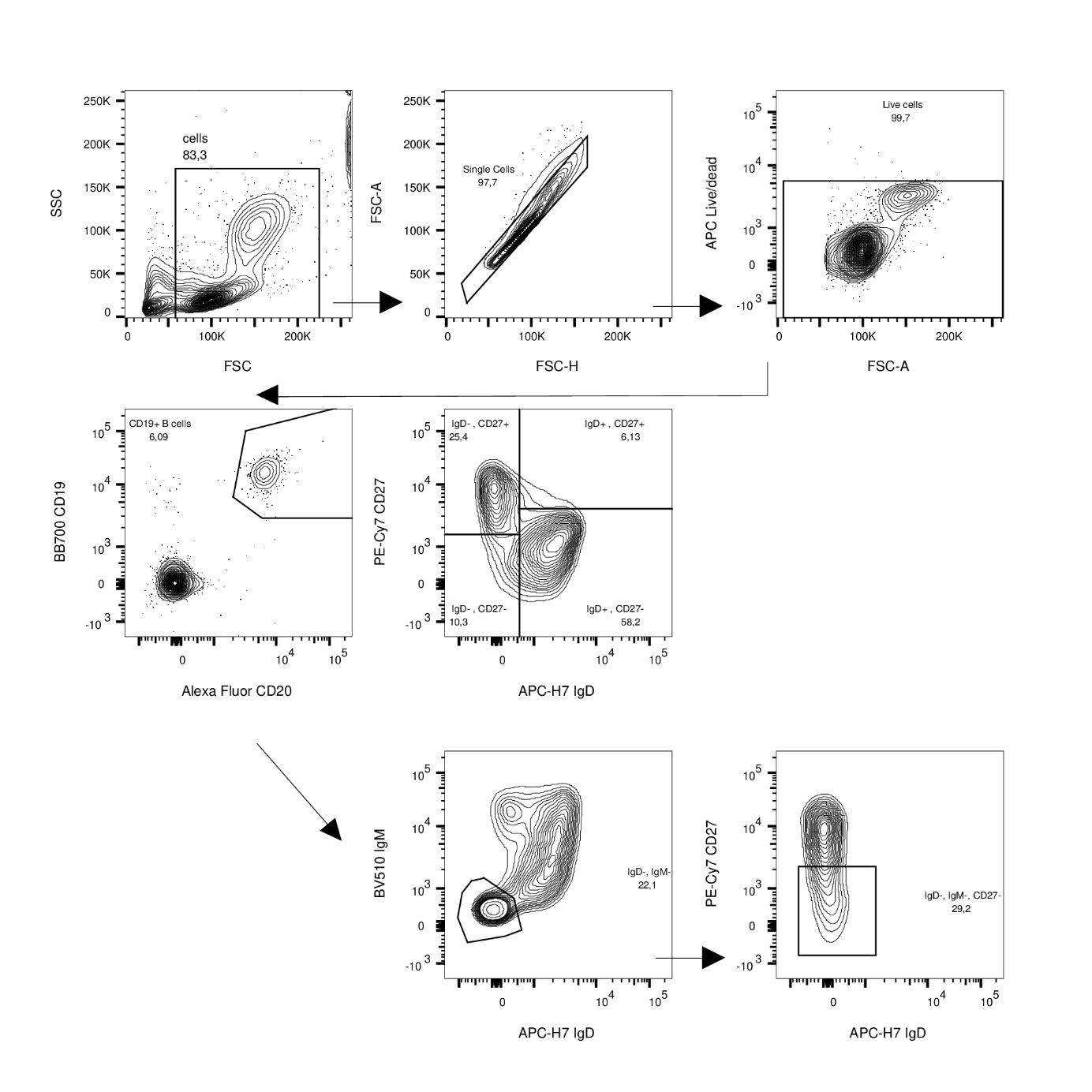
**

**Supplementary Figure 1. Gating strategy used to identify conventional and unconventional memory B cells.** B cells from PBMCs and FNAs were identified as CD19+CD20+ cells and four subpopulations were assessed: naïve (IgD+CD27-) switched conventional memory (IgD-CD27+), switched unconventional memory (IgD-CD27-), and non-switched memory (IgD+CD27+).

**
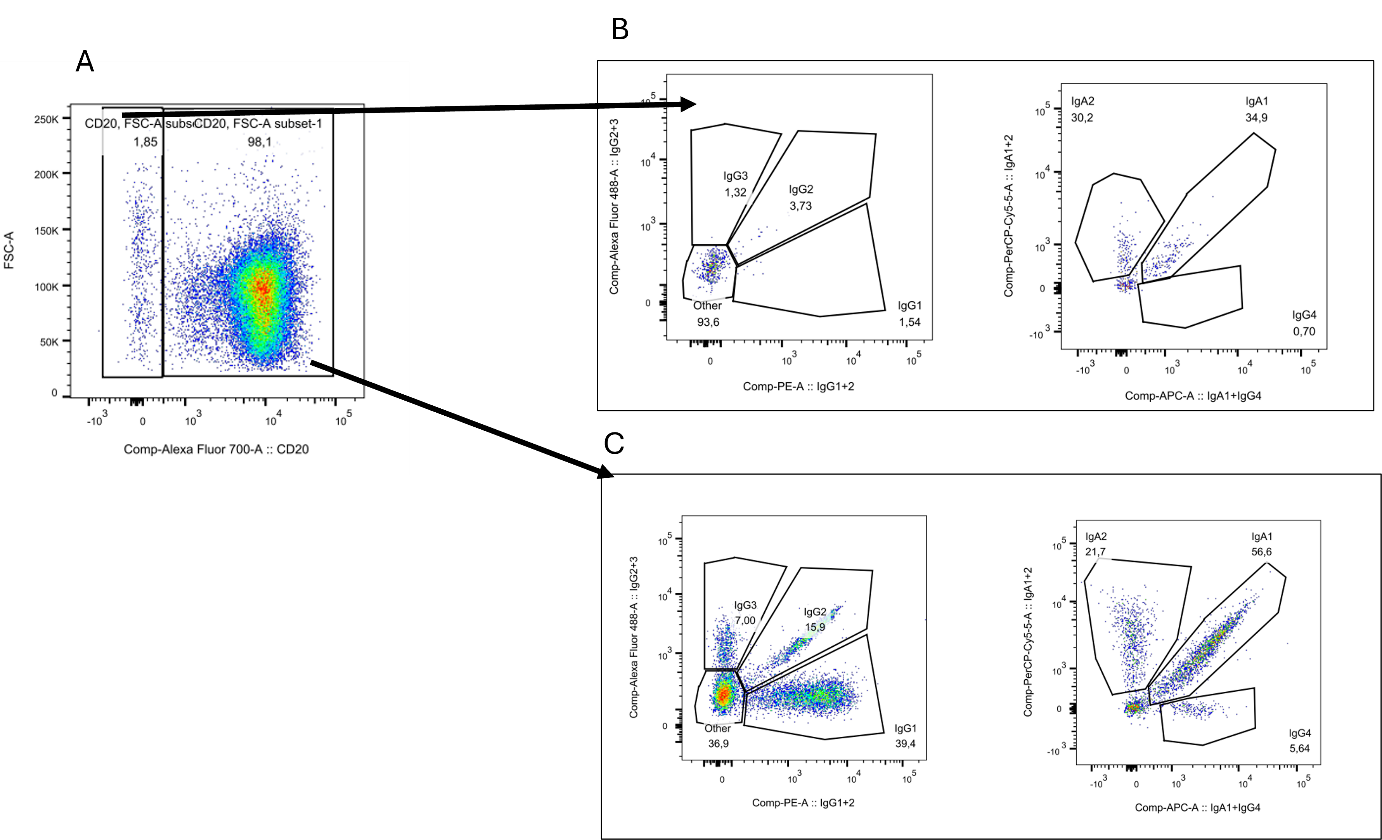
**

**Supplementary Figure 2. Gating strategy used to identify B and plasma cells expressing different IgA and IgG subclasses.** B cells and plasma cells were identified as CD19+CD20+ and CD19+CD20-, respectively, and expression of different IgA and IgG subclasses was assessed.
